# Enhanced catalytic stability for L-Dopa synthesis through cross-linking of Al₂O₃ nanocrystals with *Bacillus subtilis* tyrosine hydroxylase

**DOI:** 10.1007/s11274-025-04492-7

**Published:** 2025-07-28

**Authors:** Areesha Batool, Sikander Ali, Aneeba Rashid, Muhammad Usman Ahmad, Saba Sana, Luiza C. Campos

**Affiliations:** 1 Department of Microbiology, Dr. Ikram-ul-Haq Institute of Industrial Biotechnology, Government College University Lahore, Lahore, 54000 Pakistan; 2 Department of Botany, Dr. Nazir Ahmad Institute of Biological Sciences, Government College University Lahore, Lahore, 54000 Pakistan; 3 Centre for Urban Sustainability and Resilience, Department of Civil, Environmental and Geomatic Engineering, University College London, London, WC1E 6BT UK

**Keywords:** Al_2_O_3_ nanocrystals, Tyrosine hydroxylase, Cross-linking, Enzyme efficiency, Catalytic stability, _L−_dopa synthesis

## Abstract

This study focuses on enhancing L-Dopa synthesis by producing tyrosine hydroxylase from Bacillus subtilis SDSC-Env-i6 and its conjugation with aluminium oxide (Al₂O₃) nanoparticles. The free enzyme exhibited moderate activity, which was significantly improved through nanoparticle conjugation. Structural characterisation confirmed successful binding, with Al₂O₃ nanoparticles showing a crystalline nature and sizes ranging from 124 to 130 nm. Compared to the free enzyme, the Al₂O₃-conjugated tyrosine hydroxylase demonstrated markedly enhanced catalytic activity, reaching up to 15.3 ± 0.05 U/mL under optimised conditions. In contrast, maximum activities for the free enzyme were 5.5 ± 0.05 U/mL and 6.3 ± 0.11 U/mL in the presence of CuCl₂ and CaCl₂, respectively. The cross-linked enzyme also showed superior stability and efficiency across varying conditions. L-Dopa production was significantly higher with the conjugated enzyme, yielding between 0.531 and 1.105 mg/mL under optimised incubation conditions. These values were notably greater than those achieved using the free enzyme across different pH levels, volumes, and incubation times. Spectroscopic and microscopic analyses confirmed the integrity and functionality of the enzyme-nanoparticle complex. The observed improvements in both enzyme activity and L-Dopa yield were statistically significant (*p* ≤ 0.05), underscoring the potential of Al₂O₃ nanoparticle-conjugated tyrosine hydroxylase as an efficient and scalable biocatalyst for commercial L-Dopa production. Determination of kinetic parameters showed maximum V_max_/K_m_ (1.25 × 10^6^) was observed when immobilized tyrosinase activity was characterized with tyrosine as substrate, with significant K_cat_ value of 1.48 × 10^18^ s^− 1^.

## Graphical Abstract

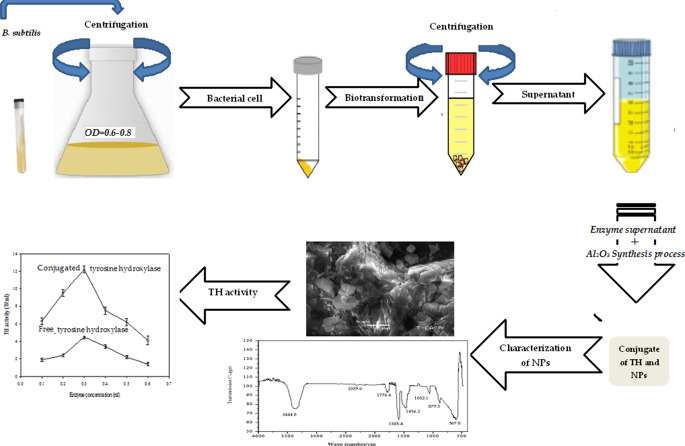

## Key points

*Bacillus subtilis* SDSC-Env-i6 produced a significant quantity of tyrosine hydroxylase, catalysing the conversion of L-tyrosine to L-Dopa, a substitute drug for Parkinson’s disease.

Al₂O₃ nanoparticles were synthesised, optimised for enzyme concentration, nanoparticle concentration, and procurement period, and successfully cross-linked with tyrosine hydroxylase.

Characterisation of the conjugated nanoparticles using UV-VIS spectroscopy, FTIR, XRD, and SEM confirmed a crystalline structure (124 to 130 nm), demonstrating enhanced enzyme activity compared to free tyrosine hydroxylase, indicating commercial potential.

## Introduction

Parkinson’s disease is the 2nd most prevalent neurodegenerative disorder affecting 2–3% of people aged above 65 years (Jiang et al. 2025). It is caused by dopamine deficiency in the brain (Labandeira-Garcia et al. 2024; Tan et al. 2021). Levodopa (3, 4-dihydroxyphenyl-L-alanine) is a gold standard therapy for Parkinson’s disease (Figura et al. 2024; Baba et al. 2022) as dopamine itself cannot cross the blood-brain barrier when administered intravenously (Lau et al. 2024; Rahman et al. 2012). In contrast, L-Dopa serves as a potent dopamine substitute that efficiently crosses the blood-brain barrier (Müller et al. 2020; Hoon et al. 2017), where it is subsequently converted to dopamine by L-aromatic amino acid decarboxylase (dopa-decarboxylase, EC 4.1.1.28) (Ledeti et al. 2017). This conversion increases dopamine levels in the brain, alleviating the symptoms of Parkinson’s disease (Min et al. 2015). The annual global demand for L-Dopa is approximately 250 metric tons, driven by the increasing prevalence of the disease (Xu et al. 2024).

Currently, L-Dopa is predominantly produced through chemical synthesis. However, this method suffers from numerous limitations, including harsh reaction conditions, low enantioselectivity, multistep processes, poor conversion rates, environmental concerns, and limited economic feasibility (Tesoro et al. 2022; Zhao et al. 2021). As a result, alternative biotechnological approaches using microbial and enzymatic methods are gaining interest. Various bacterial and fungal species, including *Aspergillus niger*, *A. ochraceus*, *Penicillium duclaauxi*, *Gliocladium deliquescens*, *Corynespora cassicola*, *Fusarium solani*, and *Trichoderma viride*, have shown potential for L-Dopa production (Min et al. 2015; Sih et al. 1969). Among bacterial species, *Escherichia coli* (Wei et al. 2016), *Halomonas bluephagenesis TD01* (Zhao et al. 2021), *E. intermedia*, *Bacillus subtilis* (Surwase et al. 2011), *Verrucomicrobium spinosum* (Tan et al. 2019), and *Erwinia herbicola* (Min et al. 2015) have also been reported as L-Dopa producers. However, microbial production remains costly due to the complexities of downstream processing and metabolite separation (Rahman et al. 2016).

Enzymatic production using tyrosinase is a more economical and sustainable alternative (Abd El-Aziz et al. 2023). Tyrosinase exhibits excellent catalytic potential for L-Dopa production and can be easily separated from the reaction medium (Nagatsu et al. 2024; Ho et al. 2003). Nevertheless, its industrial application is often hindered by poor enzyme stability and challenges in recycling (Agarwal et al. 2016). Enzyme immobilisation or conjugation onto suitable support materials offers a promising solution to these limitations (Cieńska et al. 2016). Maintaining the structural integrity and functionality of tyrosine hydroxylase, a complex enzyme, remains a challenge. The emerging field of nanotechnology provides new opportunities to address this issue (Jujjavarapu et al. 2025; Bezem et al. 2021).

Various support materials such as polyacrylamide, gelatin, chitosan-gelatin (Choi et al. 2018), alginate gel (Munjal et al. 2002), cellulose, and polystyrene microplates (Saini et al. 2014) have been employed for tyrosinase immobilisation. Recently, nano-biocatalysts have been developed by conjugating tyrosine hydroxylase onto nanomaterials, including porous silicon nanoparticles (Bezem et al. 2021) and polyhydroxyalkanoate (PHA) nanogranules (Zhao et al. 2021). Among metal oxide nanocrystals (MOx) like SiO₂, ZnO, NiO, MgO, Fe₂O₃, and Al₂O₃, aluminium oxide (Al₂O₃) stands out due to its excellent stability, biocompatibility, and surface reactivity (Hedge, 2024; Fernandez-García and Rodriguez, 2007). However, there is limited research on the conjugation of tyrosine hydroxylase with Al₂O₃ nanoparticles to enhance enzyme stability and catalytic efficiency.

The present study aimed to cross-link *Bacillus subtilis*-derived tyrosine hydroxylase with Al₂O₃ nanoparticles to improve enzyme stability and enhance catalytic efficiency for L-Dopa production. This innovative approach has the potential to offer a more stable and efficient enzymatic system for large-scale L-Dopa synthesis, with promising industrial applications.

## Materials and methods

Catalytic coating of tyrosine hydroxylase from *Bacillus subtilis* increases its stability to make it more effective for industrial utilisation. In the current study, *Bacillus subtilis-derived* tyrosine hydroxylase was coated on Al_2_O_3_ nanocrystals, and the enzyme-coated Al_2_O_3_ was characterised. Activity and stability of conjugated and free tyrosine hydroxylase were evaluated under different conditions by various analytical techniques.

### Cultivation and cell harvesting

The wild-type *Bacillus subtilis* (*B. subtilis* SDSC-Env-i6) was procured from the culture bank of the Department of Microbiology of Government College University, Lahore. The strain was revived and further maintained in a nutrient medium. For the production of tyrosine hydroxylase and _L−_dopa, the inoculum was standardised. Bacterial cell suspension was prepared by the addition of 10 mL of distilled water into *B. subtilis* slant. Inoculum was standardised (1.25 × 10^7^ CFU/mL) by counting bacterial spores using a Hemocytometer. Standardised inoculum of *B. subtilis* was inoculated into medium containing tyrosine as substrate and incubated at 37ºC for 16–18 h. After an interval of three hours, crude tyrosinase activity was measured, and cells were harvested from the culture medium. At the highest tyrosinase activity (obtained after 48 h, and OD was 0.6–0.8 at 530 nm wavelength), the culture medium was centrifuged for 15 min at 4000 rpm for harvesting of bacterial cells. The pellet was suspended in saline buffer.

### Biotransformation for tyrosine hydroxylase induction

Biotransformation is the preparatory step for the biosynthesis and extraction of tyrosine hydroxylase (TH), which is the key enzyme responsible for the subsequent conversion of L-tyrosine to L-Dopa. *B. subtilis* SDSC-Env-i6 was used for its native ability to produce TH in response to substrate induction. In this step, L-phenylalanine was used as a substrate in a reaction mixture designed to stimulate TH production. The mixture consisted of acetate buffer (10 mL, 50 mM, pH 3.5), L-ascorbic acid (5 mg/mL), and L-tyrosine (2.5 mg/mL), with a *B. subtilis* cell pellet added as a source of phenylalanine hydroxylase (PAH) and inducible enzymes. The inclusion of L-tyrosine externally served as a precursor and inducer as it selectively enhances TH production over other enzymatic pathways by mimicking the physiological presence of the substrate (Kumagai et al. 2023). The reaction was incubated at 50 °C in a shaking water bath (80 rpm) for 60 min to facilitate enzyme induction. After incubation, the mixture was centrifuged for 15 min at 3000 rpm. The resulting supernatant (8–10 mL) containing induced enzyme activity was stored at 4 °C for further experimentation (Shad et al. 2024). This experiment was conducted in duplicate. This step is essential in the methodological sequence, as the successful induction and availability of TH is a prerequisite for its subsequent conjugation with Al₂O₃ nanoparticles and application in L-DOPA synthesis.

### Determination of tyrosine hydroxylase activity

The activity of tyrosine hydroxylase produced by *B. subtilis* SDSC-Env-i6 was measured by the method of Kandaswami and Vaidyanathan (Kandaswami et al. 1973). Enzymatic supernatant (cell-free extracellular fluid obtained after centrifugation of *B. subtilis* culture post-incubation containing tyrosine hydroxylase enzyme) (0.1 mL) was added into a test tube along with 0.1 mL of _L−_ascorbic acid, EDTA and _L−_tyrosine each and 2.6 mL of phosphate buffer (pH 7.2, 50 mM) was added into it. The components were mixed thoroughly and incubated at 25 °C for 20 min. Absorbance was measured at 310 nm until it became constant. The decrease in absorbance was measured for 5 min with an interval of one minute. One unit of enzyme activity is defined as a Δ*A*_265nm_ of 0.01 per minute at 25 °C (pH 7.2) in a 3 mL reaction with _L−_ascorbic acid and _L−_catechol. The enzyme activity (TH activity U/mg) was calculated using the following formula.

1 $$\:Enzyme\:activity=\frac{\varDelta\:A310nm/\text{m}\text{i}\text{n}\left(test\right)-\varDelta\:310nm/\text{m}\text{i}\text{n}\left(control\right)\:\:\:\:}{0.01mg\:enzyme/\:reaction\:\:\:mixture\:\:\:\:\:\:\:\:\:}$$

### Stability and catalytic efficiency of tyrosine hydroxylase

The tyrosine hydroxylase stability at different temperatures was investigated by placing the enzyme reaction mixture at 20, 25, 30, 35, 45 and 65 °C and activity was recorded at each temperature. Similarly, the catalytic efficiency of the enzyme was determined using different salts of calcium (Ca(OH)_2,_ CaCl_2_ and CaSO_4_) and copper (CuSO_4_.5H_2_O, Cu(CH_3_COOH) and CuCl_2_). Among selected salts, the effect of varying concentrations (5–30 ppm) of CaCl_2_ and CuCl_2_ on enzyme activity was evaluated.

### Synthesis of Al_2_O_3_ nanoparticles

For the synthesis of Al_2_O_3_ nanoparticles, 50 mL of Al_2_O_3_ solution (1 mM) was stirred continuously for ten minutes at RT. The enzyme supernatant (10 mL) was added slowly to the above solution with continuous stirring. NaOH (1 N) was added to this reaction mixture to adjust the pH to 10. This mixture was placed for 30 min at 65 °C, and a colour change was observed from transparent (without supernatant addition) to light yellow (with addition of supernatant) and then to dark yellow. This mixture was placed in a centrifuge machine at 6,000×*g* for 20 min of centrifugation, and the pellet consisting of nanoparticles was harvested and dried at 37 °C overnight (Dutt et al. 2018).

### Catalytic coating of tyrosine hydroxylase on Al_2_O_3_-NPs

Catalytic coating was optimised using different enzyme quantity (volume of enzyme from 0.1 to 0.6 mL), concentration of Al_2_O_3_-NPs (25–300 mM/ for 0.3 mL enzyme quantity) and different procurement time (10, 20, 30, 40, 50, 60, 70 and 80 min). Al_2_O_3_-NPs with above mentioned concentration were mixed with TH at different enzyme volumes, followed by incubation at selected procurement times.

### Characterisation of Al_2_O_3_ nanoparticles

Characterization of cross-linked nanoparticles with tyrosine hdroxylase were carried out by spectroscopy using UV-VIS Digital Spectrometer (Cary 60, Agilent technologies, USA), SEM (Scanning Electron Microscopy) using electron microscope (JEOL, JSM-6480LV, Tokyo, Japan), X-ray diffraction analysis (D8 Advance, Bruker-Optik, Ettlingen, Germany) and FTIR (Spectrum-100, Perkin Elmer, St. Louis, USA). Photocatalytic estimation of Al_2_O_3_-NPs was performed using in spectrophotometric analysis. Absorption spectrum was obtained at the light spectrum of 200–800 nm after sonication of synthesised Al_2_O_3_-NPs in distilled water for five minutes (Piriyawong et al. 2012). In FTIR analysis, the absorption spectrum of dry and refined Al_2_O_3_-NPs was evaluated by using an attenuated total reflection (ATR) mode (Ismail et al. 2021). In this analysis, photoconductivity, infrared (IR) spectra of emission and immersion of Al_2_O_3_-NPs were acquired. The sample was spread on a silicon wafer to form a coated film. Absorption spectrum was recorded in the range of 400–4000 cm^− 1^.

The size of the crystallite and crystallinity were determined by XDR analysis (Nila et al. 2018). Structure and phase components of synthesised nanoparticles of Al_2_O_3_ were determined using monochromatized Cu Kα radiation (λ = 0.154 nm) with diffraction angle range 2θ = 5–90° and scan rate 0.05/min. Diffraction peaks created a characteristic X-ray diffraction spectrum of Al_2_O_3_-NPs (Bunaciu et al. 2015). The morphology (size and shape) of nanoparticles was determined by scanning electron microscopy as mentioned by Ramlee et al. (2022).

### Evaluation of *B. subtilis*_L−_Dopa production

_L−_Dopa was estimated by following the Arnow method (1937). The enzyme supernatant (1 mL) was added to the test tube using a sterilised pipette. An equal volume of HCl (0.5 N) and nitrite molybdate was added and mixed properly. After the appearance of yellow colour, 1 mL of NaOH (1 N) was added and the solution was properly mixed. When the mixture turned red, distilled water was added to the mixture to bring the final volume up to 5 mL. Again, the contents of the test tube were mixed well and placed at 30 °C for 05 min. The experiment was conducted in duplicate. A blank was prepared with the same procedure using distilled water (one mL) instead of supernatant. Absorbance (OD value) was measured at 530 nm against a blank using a spectrophotometer (VS-1100, BMS, Europe). The _L−_dopa was quantified (mg/mL) using the equation (L-dopa = X/5) and value of X derived from the standard curve (X = y + 0.0005/1.9945). Where y is the absorbance (OD value) measured by the procedure assay at 530 nm, and 5 is the total volume of the reaction mixture.

### Determination of Dopa decarboxylase activity

The dopa decarboxylase activity was measured using the method of Sherald et al. (1973). The enzyme supernatant (0.05 mL) was added into a test tube along with 0.1 mL of 5 µM pyridoxal-5ˊ-phosphate and 0.1 mL of _L−_dopa (5 µM) solution. After 30 min of incubation at 37 °C, the contents were mixed properly, followed by the addition of one mL of potassium cyanide (KCN) solution (0.1%). The reaction mixture was placed at 37 °C for 20 min of incubation, followed by the addition of 1.5 mL of benzene. The contents were vortexed for 15 s, and the reaction tube was centrifuged for 15 min at 4000 rpm. The upper phase was separated, and absorbance was measured at 410 nm against a blank. One unit of enzyme activity is a related amount of enzyme that accelerates the decarboxylation of one micromole of _L−_dopa in 30 min. The enzyme activity was expressed as µmol (Tate et al. ).

### Integrated Inhibition of Dopa decarboxylase activity

Two potential inhibitors were chosen for comparative integrated inhibition of dopa decarboxylase (Aromatic Amino Acids decarboxylase) activity (Leyden and Tadi 2025; Beckers et al. 2022; Müller 2012; Jonkers et al. 2001). The inhibitors were benserazide and carbidopa. The effect of different concentrations (5 to 30 ppm) and exposure time (post addition) of benserazide and carbidopa, and incubation temperature (20, 25, 30, 35, 45 and 65 °C) was studied on dopa decarboxylase activity.

### Kinetic studies

Double Reciprocal Lineweaver-Burl plot was used to estimate K_m_ (Michaelis-Menten constant) and V_max_ (maximum reaction velocity) values of enzyme for all substrates as described by Lineweaver and Burk (1934). Graph was plotted between reciprocal of V (reaction velocity, calculated according to Eq. 2) and reciprocal of used substrates. Following equation of straight line from plot was referred to Lineweaver-Burk equation to calculate kinetic parameters (Bennett and Frieden 1969).

$$\:\frac{\text{1}}{\text{V}}\text{=\:}\frac{{\text{K}}_{\text{m}}}{{\text{V}}_{\text{max}}}\left(\frac{\text{1}}{\text{S}}\right)\text{+}\frac{\text{1}}{{\text{V}}_{\text{max}}}$$

Enzyme turn over number was estimated by following equation:

$$\text{K}_{\text{cat}}\text{=\:}\frac{\rm{No.\,of\,molecules\,of\,enzyme\,used\,to\,calculate\,V_{max}\,(x,M/s)}}{\rm{amount\,of\,enzyme\,in\,x,M}}$$

Reusability of immobilized enzyme.

To assess the reusability of an immobilized enzyme, the immobilized enzyme in a reaction cycle was repeatedly using and its activity was determined over time under standard reaction conditions. The enzyme’s activity was checked after each cycle and comparing it to its initial activity, allowing for the determination of residual activity.

### Statistical analysis

Treatments were compared using ANOVA using SPSS (version 20), followed by post-hoc and protected least significant difference method (Snedecor and Cochran 1980). Duncan’s multiple ranges in the form of probability < ρ > values were used to represent significant differences among.

## Results

*B. subtilis* SDSC-Env-i6 was evaluated for tyrosine hydroxylase. _L−_dopa production in different harvesting volumes from culture medium, varying the pH of culture medium and incubation time was also measured. Catalytic coating of tyrosine hydroxylase from *B. subtilis* SDSC-Env-i6 with AL_2_O_3_ has been investigated, and it resulted in a stable and highly active tyrosine hydroxylase for the production of _L_-dopa.

### Evaluation of tyrosine hydroxylase thermostability

**Fig. 1:**
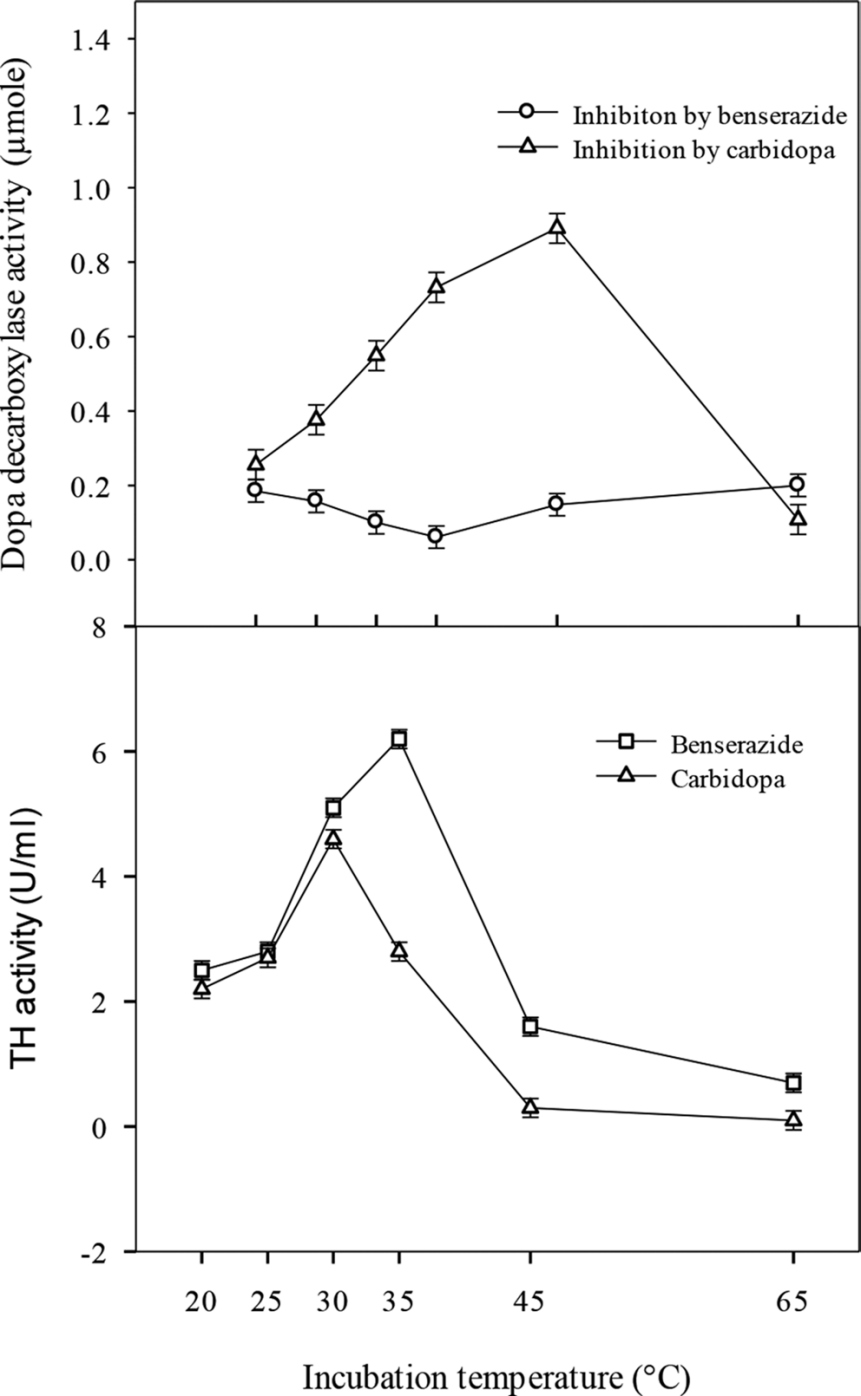
Effect of incubation temperature on dopa decarboxylase activity and tyrosine hydroxylase thermostability

Tyrosine hydroxylase was produced from *B. subtilis* SDSC-Env-i6 by submerged fermentation. Different incubation temperatures (20, 25, 30, 35, 45 and 65 °C) along with two benserazide and carbidopa were utilised for the determination of tyrosine hydroxylase thermostability and AAA decarboxylase activity. According to results, the least enzyme activity of AAA decarboxylase by benserazide (0.061 ± 0.01 µmole) and carbidopa (0.093 ± 0.05 µmole) was determined at 35 °C and 65 °C, respectively. A 0.65-fold decreased activity was observed with benserazide in comparison with carbidopa (Fig. 1). Moreover, the activity of tyrosine hydroxylase was highest (4.6 ± 0.09 U/mL) at 30 °C for carbidopa. However, the highest enzyme activity was benserazide with 6.2 ± 0.01 U/mL at 35 °C. A 1.3-fold increase in activity was observed with benserazide as compared to activity with carbidopa. However, there was a sharp decrease in activity with a further temperature rise, as shown in Fig. 1.

### Evaluation of tyrosine hydroxylase catalytic efficiency

**Fig. 2:**
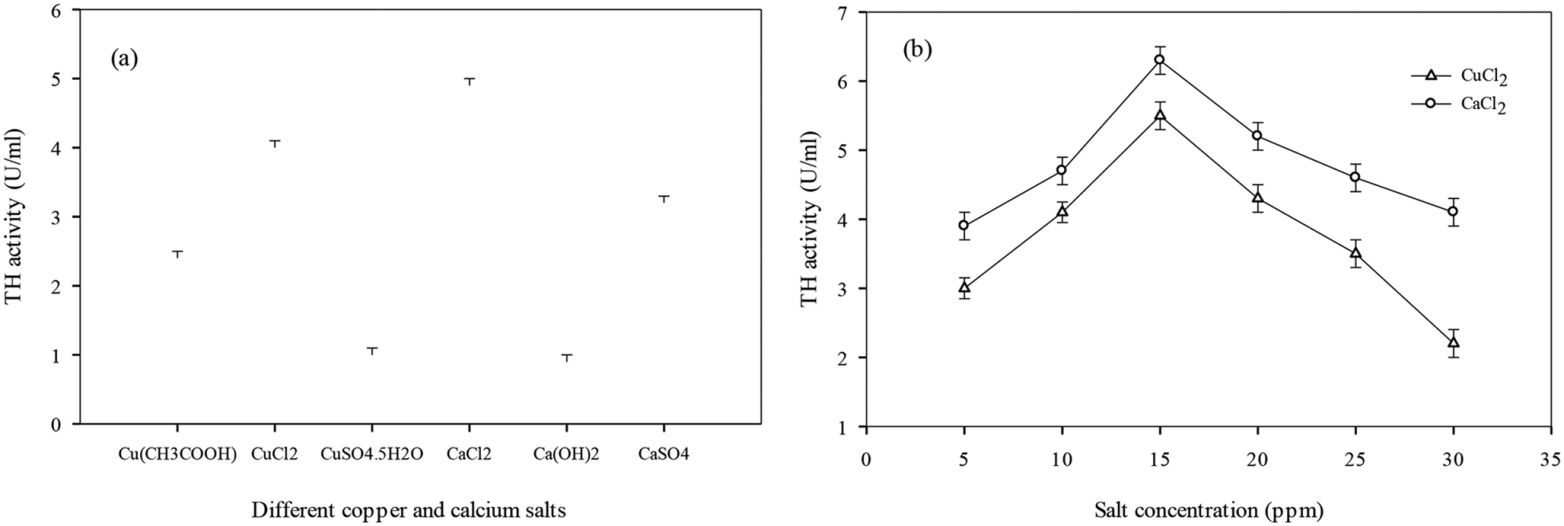
Effect of different concentrations of copper and calcium salt on tyrosine hydroxylase catalytic efficiency

Copper and calcium salts in different concentrations were evaluated for their effect on improving tyrosine hydroxylase catalytic efficiency. Three copper salts (CuSO_4_.5H_2_O, CuCl_2_ and Cu (CH_3_COOH)) and three calcium salts (CaCl_2_, Ca (OH)_2_ and CaSO_4_) with varying concentrations (5, 10, 15, 20, 25 and 30 ppm) were observed for their effect on enzyme activity. Among selected salts, the highest activity was found with CuCl_2_ (5.1 ± 0.07 U/mL) and CaCl_2_ (4.6 ± 0.03 U/mL). According to the results of the concentration effect, the highest activity was 5.5 ± 0.05 U/mL for CuCl_2_ and _6_.3 ± 0.11 U/mL for CaCl_2 at_ 15 ppm. The lowest activity for CuCl_2_ was 3 U/mL at 5 ppm and 4.1 U/mL for CaCl_2_ at 30 ppm. The results are presented graphically in Fig. 2.

### Catalytic coating of tyrosine hydroxylase on Al_2_O_3_-NPs

TH obtained from *B. subtilis* SDSC-Env-i6 was coated on Al_2_O_3_-NPs to enhance the stability and ultimately improve efficiency. The coating was optimised using different volumes of the enzyme-containing supernatant, concentration of nanoparticles and procurement time after mixing. Tyrosine hydroxylase activity was measured and compared in coated form and alone.

### Volume optimisation of enzymatic supernatant for Al_2_O_3_-NPs conjugation

**Fig. 3:**
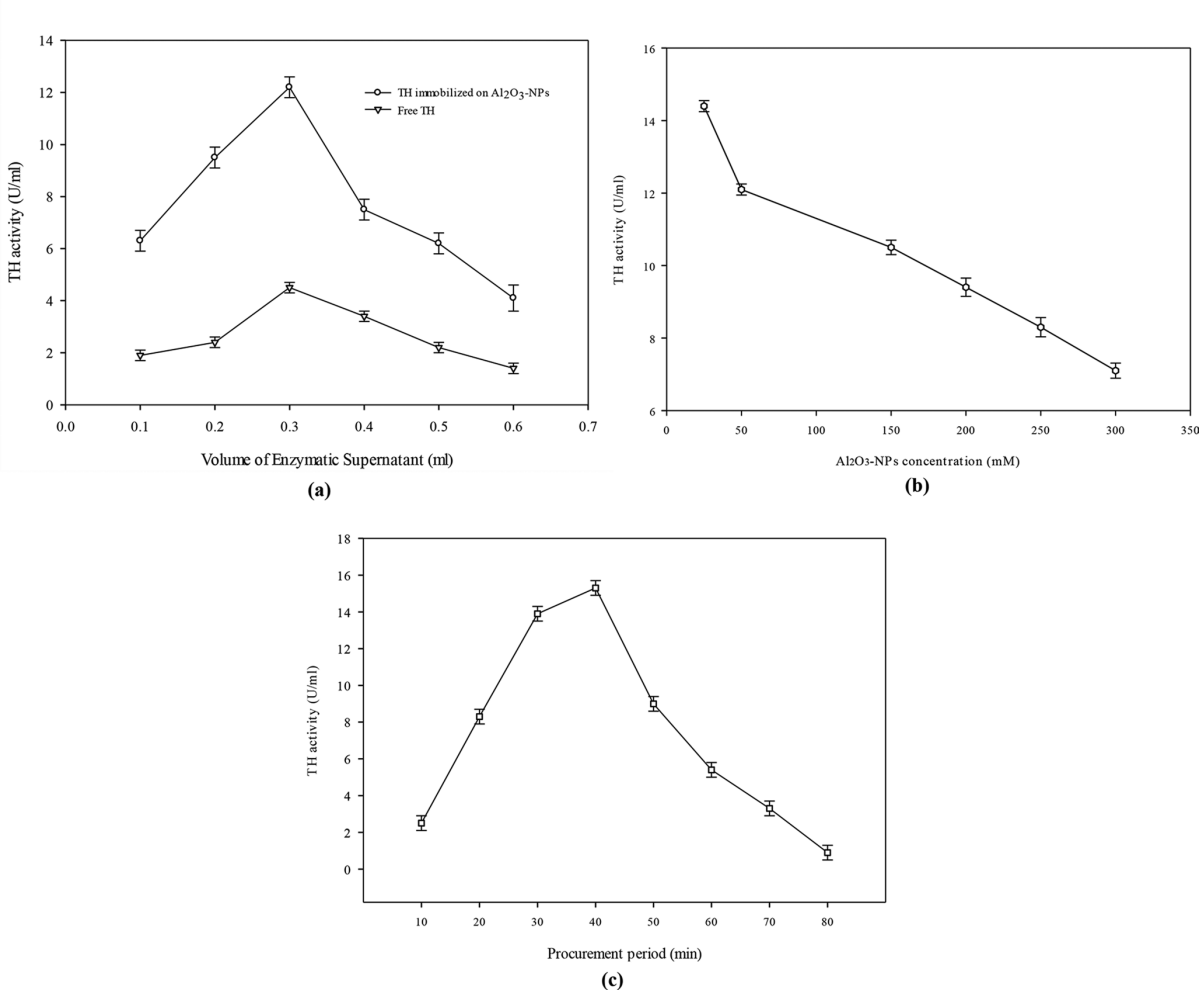
(**a**) Effect of different volumes of enzymatic supernatant for the coating of tyrosine hydroxylase on Al_2_O_3_-NPs. (**b**) Effect of different Al_2_O_3_-NPs concentrations for coating of tyrosine hydroxylase. (**c**) Effect of different procurement times for the coating of tyrosine hydroxylase on Al_2_O_3_-NPs

The different volumes of the enzymatic supernatant containing tyrosine hydroxylase (0.1, 0.2, 0.3, 0.4, 0.5 and 0.6 mL) were used for investigation of tyrosine hydroxylase conjugation on Al_2_O_3_-NPs. Free and conjugated enzyme activity was measured and compared. The results are presented in Fig. 3a. Free and conjugated enzymes showed the least activity with 0.2 mL and 0.6 mL of enzyme volume, respectively. However, the highest activity was recorded using 0.3mL of free tyrosinase in free as well as in conjugated form. But conjugated enzyme activity was higher (12.1 ± 0.12 U/mL) as compared to free TH (4.5 ± 0.07 U/mL) with the same volume. Enzyme activity was 2.7-fold higher in the conjugated form. Initially, a linear relationship was observed between enzyme volume and activity of conjugated tyrosinase at low volume of enzyme volume. But after 0.3 mL enzyme volume, 4-fold less activity was observed on using 0.6 mL of the conjugated form of the enzyme. A similar trend was observed for free enzyme. A 1.3-fold decline was recorded using an enzyme volume higher than 0.3 mL.

### Optimisation of Al_2_O_3_-Nps concentration

Different Al_2_O_3_-NPs concentrations (25, 50, 150, 200, 250 and 300 mM) were used for tyrosine hydroxylase coating. The activity was recorded at each concentration. However, the highest enzyme activity (14.4 ± 0.15 U/mL) was recorded with a 25 mM concentration of Al_2_O_3_-NPs. A two-fold increase in the activity of the enzyme was observed with 25 mM concentration in comparison to 300 mM of Al_2_O_3_-NPs. There was an indirect relation between enzyme activity and Al_2_O_3_-NPs concentration as shown in Fig. 3b.

### Optimisation of procurement period

The effect of different procurement times (10, 20, 30, 40, 50, 60, 70 and 80 min) for the conjugation of tyrosine hydroxylase on Al_2_O_3_-NPs was observed. The enzyme activity was measured at each procurement period. The highest activity (15.3 U/mL ± 0.05) was recorded after 40 min of procurement. There was a 6.1-fold higher activity was at 30 min as compared to 10 min of procurement. Reduced activity was observed with increase in adsorption time after 40 min of procurement. Longer procurement time increases the adsorption of enzyme until adsorption equilibrium is achieved as shown in Fig. 3c. However, after adsorption equilibrium, increase in procurement period results in decreased activity of enzyme.

### Characterisation of Al_2_O_3_-NPs

The Al_2_O_3_-NPs were synthesised and using for catalytic coating of tyrosine hydroxylase. UV-VIS Spectrophotometry, FTIR, X-ray diffraction and scanning electron microscopy were used for Al_2_O_3_-NPs coated with tyrosine hydroxylase.

### UV-VIS spectrophotometry

**Fig. 4:**
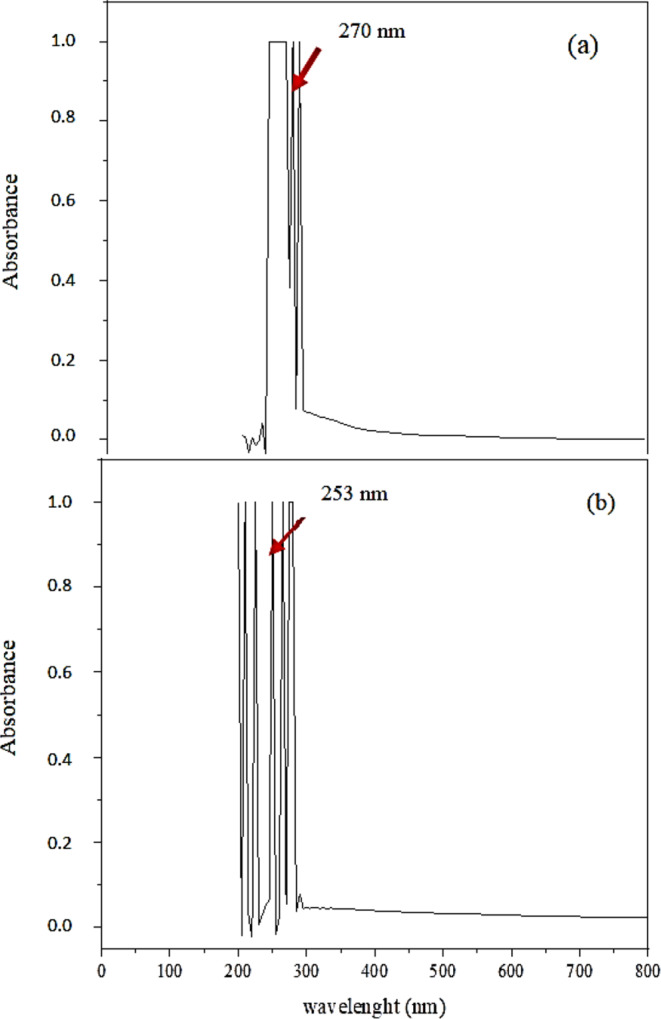
**(a)** UV-VIS absorption spectrum of free tyrosine hydroxylase, **(b)** UV-VIS absorption spectrum of conjugated tyrosine hydroxylase with Al_2_O_3_

Wavelengths of light between 200 and 800 nm were used for analysis of homogenously dispersed Al_2_O_3_-NPs solution. The absorption spectrum of nanoparticles was recorded with a double-beam beam at room temperature. The scan rate was 24,000 nm/min. Absorption spectrum of Al_2_O_3_-NPs is shown in Fig. 4. The highest peak in the absorption spectrum was recorded at 253 nm.

### Fourier transform infrared spectroscopy (FTIR)

**Fig. 5:**
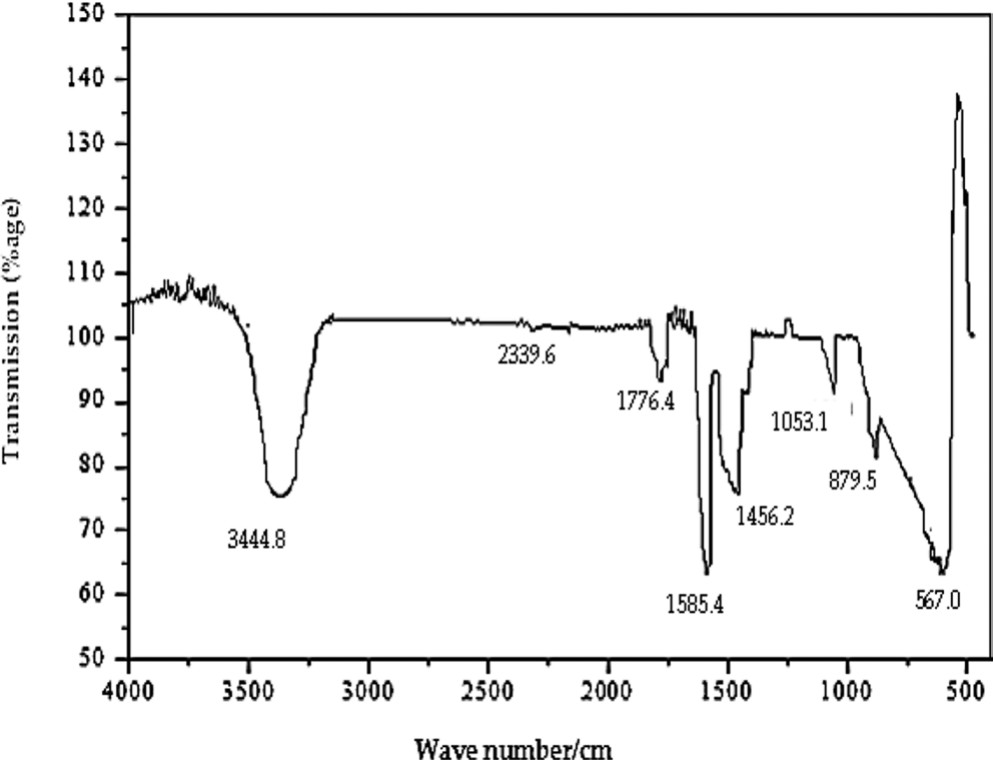
FTIR spectrum of chemically synthesised and immobilized enzyme–nanoparticle complex of Al_2_O_3_-NPs

One of the reliable and dynamic quantitative analytical techniques is FTIR spectroscopy. This technique provides significant information about the structure, which may not be achieved by other methods. The present study deals with the use of FTIR analysis for the identification of stabilising functional groups adsorbed on the Al_2_O_3_-NPs surface. FTIR spectroscopy of Al_2_O_3_-NPs is presented in Fig. 5. The FTIR spectrum of Al_2_O_3_-NPs was recorded between a scanning range of 400–4000/cm. A strong absorption band was observed between 520–900/cm. Due to Al-O-Al stretching vibration, FTIR peaks at 567 cm^− 1^ and 879 cm^− 1^ were observed.

The absorption peak at 3444 cm^− 1^ corresponds to the O-H stretching vibration. However, the absorption peak recorded at 2339 cm^− 1^ was due to the stretching vibration of the N-H bond, which is the Al_2_O_3_-NPs associated functional group. The C = O functional group produced a strong absorption band at 1585 cm^− 1^. The bending vibration of the C-O functional group was detected at 1053 cm^− 1^.

### X-Ray diffraction (XRD)

**Fig. 6:**
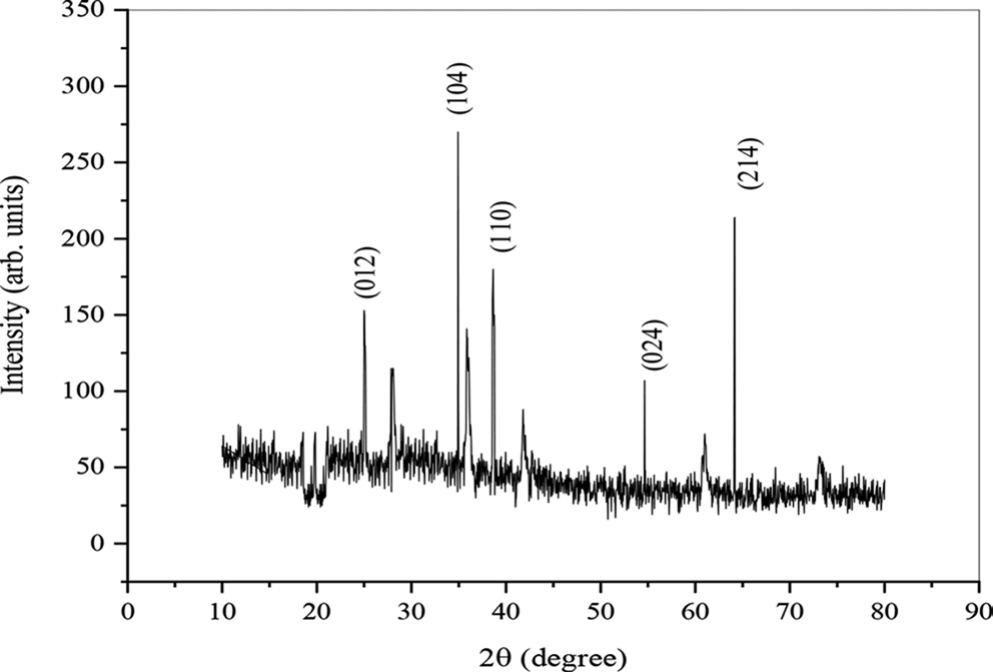
XRD patterns of chemically synthesised Al_2_O_3_-NPs

In this study, XRD was used for the determination of the crystalline nature of synthesised Al_2_O_3_-NPs. The instrument operation is based on continuous scanning in a vast range of angles. Each step was carried out for 0.2 s, and the scanning step was 0.02°/sec. The diffraction equipment is equipped with a copper anode. This anode has a K_α_ of 0.1540598 nm wavelength. The profile of Al_2_O_3_-NPs obtained in VXDR is presented in Fig. 6. High-intensity diffraction peaks were obtained in this pattern. Peak indexing was performed using Powder X software. The peak obtained was similar to the record of alumina structure stored in JCPDS card number 42-1468. At 25.32° (012), 34.96° (104), 38.15° (110), 54.59° (024) and 64.29° (214) sharp diffraction peaks were observed. These confirmed the Al2O3-NPs’ crystalline nature. The intensity peaks were observed with small impurities, and other minor peaks were representative of noise.

### Scanning electron microscopy (SEM)

**Fig. 7:**
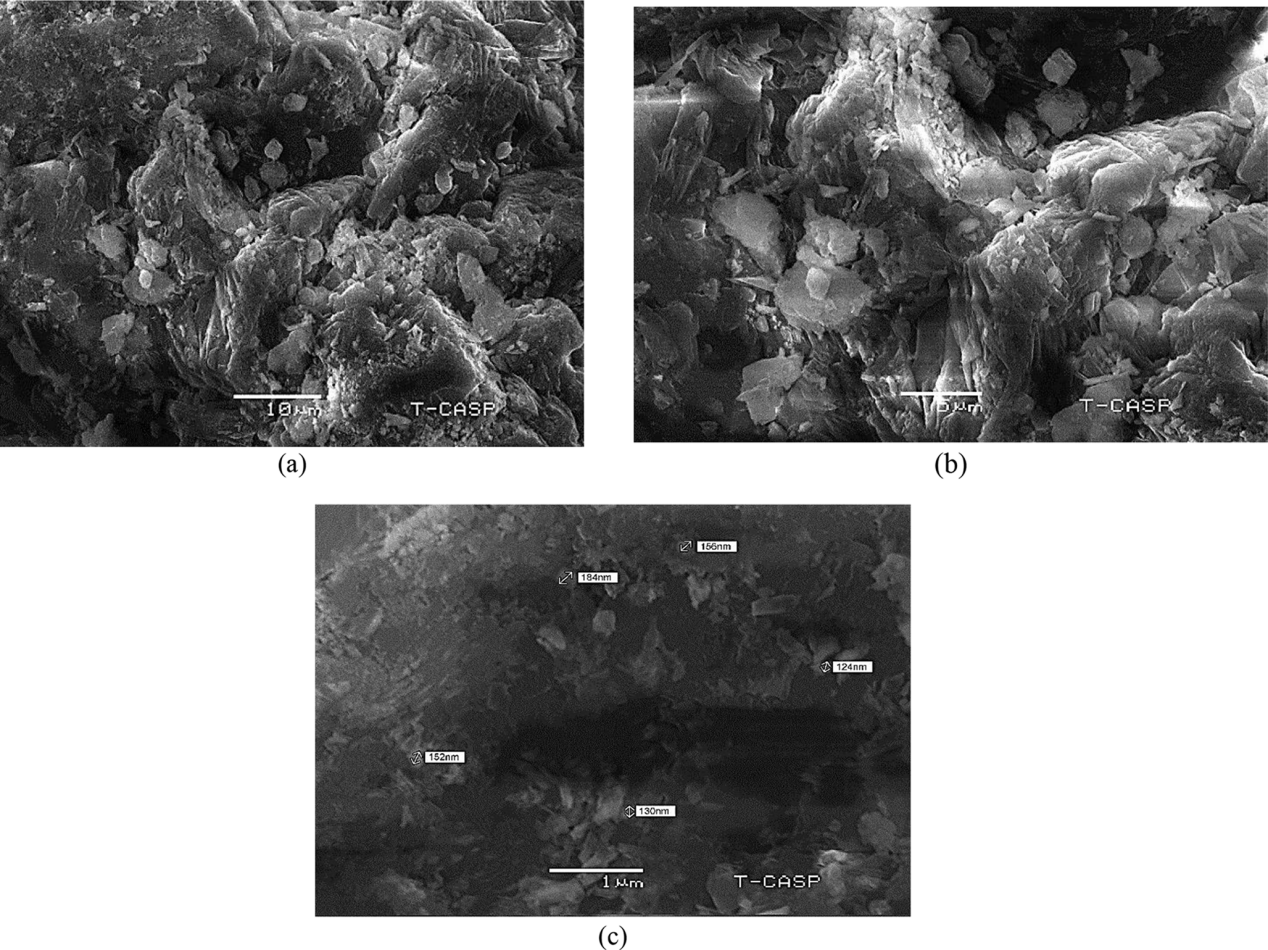
(**a**) SEM micrograph of Al_2_ O_3_ nanoparticles at 50 μm; (**b**) SEM micrograph of Al_2_ O_3_ nanoparticles at 10 μm; (**c**) SEM micrograph of Al_2_ O_3_ nanoparticles at 1 μm

Different magnifications (50, 5 and 1 μm) were used in SEM for Microstructural analysis of Al_2_O_3_-NPs as presented in Fig. 7. These SEM images confirmed the irregular spherical shape and crystalline formation of Al_2_O_3_-NPs. At 1 μm magnification, the particle size ranged from 124 to 130 nm. Some agglomerated particles at 156 nm and 184 nm were also observed in the SEM micrograph.

### Evaluation of *B. subtilis* SDSC-Env-i6 for _L−_Dopa production

The effect of harvesting medium volume on the production of _L−_dopa using SDSC-Env-i6 of *B. subtilis* was evaluated. Results are presented in Table 1. Cells were harvested from 25, 50, 75, 100, 125 and 150 mL of culture medium. Higher cell mass was obtained in 50 mL and 75 mL of culture medium. However, _L−_dopa production from the cell pellet collected from 50 mL was 1.6-fold higher from the pellet obtained from 75 mL of medium. Cell pellet harvested from 50 mL of medium produced the highest quantity of _L−_dopa (0.522 ± 0.002 mg/mL). However, the production of _L−_dopa decreased with the increase in volume of harvesting medium beyond 75 mL.

Different pH (5, 5.5, 6, 6.5, 7, and 7.5) values were evaluated for their effect on the _L−_dopa production using SDSC-Env-i6 of *B. subtilis.* Results are shown in Table 1. Larger cell pellets were harvested at pH 6 and 6.5. _L−_Dopa production increased with the gradual increase in pH value of the cultivation medium from 5 to 6.5. The highest _L−_dopa production (0.897 ± 0.01 mg/mL) was observed at pH 6.5. A 5.5-fold increase in _L−_dopa production was obtained at pH 6.5 in comparison to pH 5.0. _L−_dopa production decreased with further increase in pH beyond 6.5. The highest production of _L−_dopa at pH 6.5 could be linked to the higher metabolic rate at this pH.

The effect of incubation time on _L−_dopa production by *B. subtilis* SDSC-Env-i6 was evaluated as mentioned in Table 1. The culture medium was collected after 12, 24, 48, 72, 96, and 144 h of incubation to get cell mass. However, 48 h was the optimal time of incubation as the highest _L−_dopa quantity (1.104 ± 0.007 mg/ml) was observed. There was 2.1-fold higher production at 48 h as compared to 12 h incubation. A gradual decrease in production was observed at longer incubation times (after 48 h).

**Table 1:** _L−_Dopa production from *B. subtilis* SDSC-Env-i6

Effect of harvesting medium pH on _L−_dopa production		Effect of harvesting medium volume on _L−_dopa production		Effect of incubation time on _L−_dopa production	
pH	(mg/mL)	Volume (mL)	mg/mL	Time (h)	mg/mL
5	0.163	25	0.203	12	0.531
5.5	0.484	50	0.523	24	0.635
6	0.732	75	0.315	36	0.897
6.5	0.897	100	0.266	48	1.105
7	0.685	125	0.112	60	0.902
7.5	0.345	150	0.096	72	0.724

### Determination of kinetic parameters

**Fig. 8:**
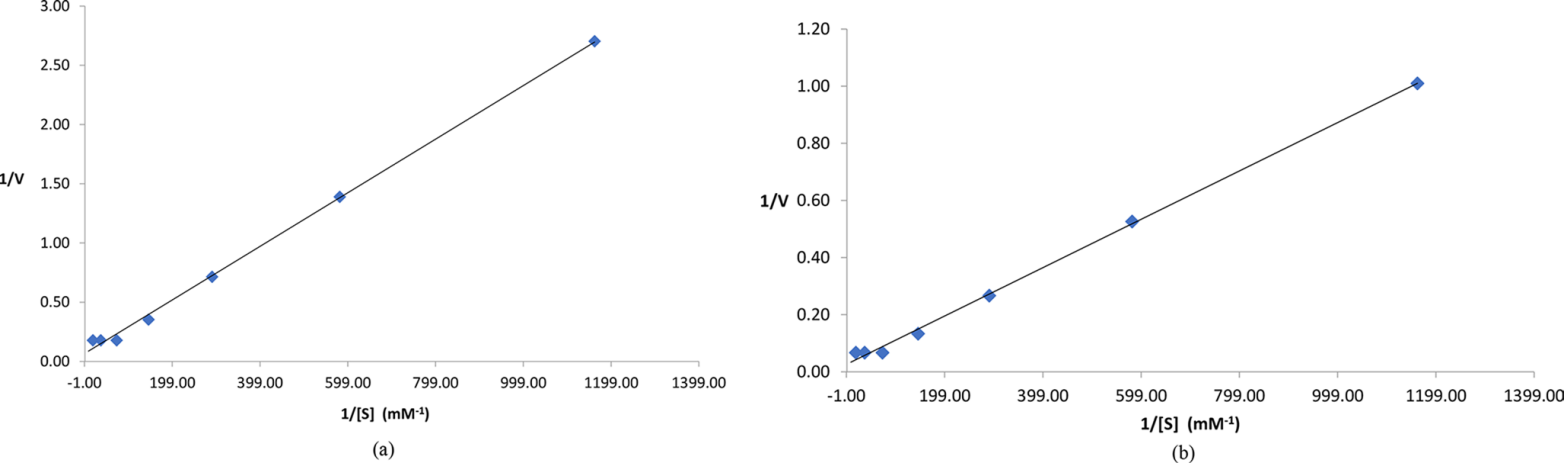
(**a**) Double Reciprocal Lineweaver-Burk plot for free tyrosine hydroxylase with different concentrations of L-tyrosine as substrate. (**b**) Double Reciprocal Lineweaver-Burk plot for free immobilized hydroxylase with different concentrations of L-tyrosine as substrate

Figure 8 (a) and (b) shows Lineweaver-Burk plot for free and immobilized tyrosine hydroxylase at standard conditions. K_m_ values of 3.35 × 10^− 5^ M^− 1^ (with V_max_ 14.56) and 2.97 × 10^− 5^ M^− 1^ (with V_max_ 37.17) was observed for free and immobilized enzyme preparations respectively with different concentrations of substrate (Table 2). Very efficient enzyme turn over number with K_cat_ value of 1.40 × 10^18^ s^− 1^ was also observed for immobilized enzyme.

**Table 2:** Effect of various concentrations of substrate on activity of free and immobilized enzyme preparations

L-Tyrosine (Substrate Conc.) mg/mL	L-Tyrosine (Substrate Conc.) mM	Free L-Tyrosine Hydroxylase (U/mL)	Immobilized L-Tyrosine Hydroxylase (U/mL)	1/[S]	1/V (Free Enzyme)	1/V (Immobilized Enzyme)
0.156	8.61 × 10^− 4^	0.37	0.99	1161.47	2.70	1.01
0.312	1.72 × 10^− 3^	0.72	1.9	580.74	1.39	0.53
0.625	3.45 × 10^− 3^	1.4	3.75	289.90	0.71	0.27
1.25	6.90 × 10^− 3^	2.83	7.48	144.95	0.35	0.13
2.5	1.38 × 10^− 2^	5.62	14.87	72.48	0.18	0.07
5	2.76 × 10^− 2^	5.62	14.87	36.24	0.18	0.07
10	5.52 × 10^− 2^	5.62	14.87	18.12	0.18	0.07

### Enzyme reusability

Immobilized tyrosine hydroxylase showed significant reusability upto five cycles with only 28.12% reduction in activity (Table 3).

**Table 3:** Determination of reusability of immobilized TH

Reuability cycles	Activity Loss (%) of Immobilized Tyrosine Hydroxylase
1	5.5
2	13.62
3	18.45
4	23.56
5	28.12
6	35.05
7	58.24
8	76.86

## Discussion

In the current study, the activity of tyrosine hydroxylase was found to be highest (4.6 ± 0.09 U/mL) at 30 °C in the presence of carbidopa. Elevated temperatures beyond this optimum likely led to enzyme denaturation, consistent with the findings of Daniel et al. (2007). In contrast to current data, Surwase et al. (2011) documented the highest enzyme activity at 40 °C, indicating variability depending on the microbial strain or experimental conditions. In contrast to our results, Agarwal et al. (2016) and Tan et al. (2021) reported stability of tyrosine hydroxylase at 55 and 50 °C, respectively. These discrepancies suggest organism-specific responses and different enzyme isoforms. The advantage of levodopa/decarboxylase inhibitor (DDI) co-therapy lies in the ability of DDIs such as carbidopa and benserazide to prevent peripheral metabolism of L-dopa, thereby facilitating its transport to the central nervous system (CNS), as these inhibitors do not cross the blood–brain barrier (Daidone et al. 2012; Hinz et al. 2021). This reduces the required dosage of levodopa while achieving therapeutic efficacy (Hinz et al. 2021).

The influence of various metal salts on enzyme activity was also evaluated. Among the tested copper and calcium salts, CuCl₂ and CaCl₂ exhibited the highest enzyme activities (5.1 ± 0.07 U/mL and 4.6 ± 0.03 U/mL, respectively). This could be attributed to the role of copper as a cofactor in tyrosine hydroxylase, enhancing its catalytic function, as reported by Surwase et al. (2011). However, increased salt concentrations beyond optimal levels led to reduced activity, possibly due to the synthesis of oxidised by-products or metal toxicity (Tan et al. 2021). Similarly, Iuvone (1984) noted that low concentrations of CaCl₂ act as enzyme activators. Contrary to our findings, Surwase et al. (2011) found the highest enzyme activity with CuSO₄ at 0.04 g/L, while Tan et al. (2021) documented optimal activity with 0.06 ppm of copper ions. Iuvone (1984) also reported enzyme activation at 0.04 ppm of CaCl₂, differing from the concentrations used in the present study.

Enzyme immobilisation using aluminium oxide nanoparticles (Al₂O₃-NPs) significantly enhanced the activity of tyrosine hydroxylase. A higher enzymatic activity (12.1 ± 0.12 U/mL) was observed in the conjugated form compared to the free enzyme (4.5 ± 0.07 U/mL) at the same enzyme concentration, indicating successful adsorption and increased catalytic efficiency. This is likely due to the saturation of enzyme molecules on nanoparticle surfaces, enhancing stability and reusability (Khoshnevisan et al. 2017). Immobilised enzymes are known to retain higher functional efficiency and operational stability over time20 (Ahmad and Sardar 2015). Nonetheless, higher enzyme or nanoparticle concentrations led to agglomeration, reducing the number of available active sites (Khoshnevisan et al. 2017).

Moreover, excessive nanoparticle concentrations exhibited an inhibitory effect on enzyme function, potentially by blocking active enzyme sites or altering surface chemistry (Mishra et al. 2021). Supporting this, Liu et al. (2017) reported optimal tyrosine hydroxylase activity with 10 mM aminated magnetic nanoparticles (Fe₃O₄-NH₂), while Ziva et al. (2021) observed optimal results using 0.5 mM and 10 mM Al₂O₃-NPs. Husain et al. (2011) also demonstrated maximum enzyme activity at 100 mg ZnO-NP concentration, reinforcing the concentration-dependent response.

In the current investigation, the adsorption of undesired proteins or impurities onto nanoparticles may have reduced enzyme availability and stability, a phenomenon similarly described by Mishra et al. (2021). The effect of procurement time on enzyme activity revealed that maximum activity was attained at 40 min of conjugation, in agreement with Liu et al. (2017). However, other studies have reported differing optimal times: Bussamara et al. (2012) observed highest activity at 150 min, while Yasutaka et al. (2011) reported optimal activity at 12 h. These variations could be due to differences in experimental conditions or microbial strains used.

For the characterisation of Al₂O₃-NPs, UV-VIS spectrophotometric analysis revealed a maximum absorbance peak at 253 nm, indicative of nanoparticle formation. This is consistent with findings by Ismail et al. (2021), who reported peaks at 255 nm and 260 nm. However, alternative peaks have also been reported at 210 nm (Piriyawong et al. 2012) and 238 nm (Prashanth et al. 2015), likely due to differences in synthesis methods or nanoparticle morphology.

FTIR analysis displayed a strong absorption band in the fingerprint region (520–900/cm), confirming characteristic Al–O bond vibrations. Observed peaks align with those reported by Nila and Radha (2018), including functional groups such as O–H, C–O, and Al–O–Al. Similarly, Ramlee et al. (2022) identified peaks at 495, 588, and 723 cm^−^¹ representing bending and stretching modes.

XRD analysis further confirmed the crystalline nature of Al₂O₃-NPs, with diffraction peaks corresponding to standard Miller indices, particularly (104), indicating a preferential crystalline orientation (Bhoi et al. 2019). The current findings also align with patterns observed by Nila and Radha (2018) and Prashanth et al. (2015), validating the formation of aluminium oxide nanoparticles.

SEM imaging revealed that Al₂O₃-NPs displayed irregular spherical shapes with a particle size distribution between 124 and 130 nm. Minor agglomerates measuring 156 nm and 184 nm were also visible. These results are comparable to those of Bhoi et al. (2019), who reported particle sizes of 24 nm, and Ismail et al. (2021), who observed sizes ranging from 50 nm to submicron levels. SEM micrographs by Rajaeiyan and Bagheri-Mohagheghi (2013) showed spherical and irregular hexagonal shapes of Al₂O₃ particles synthesised through sol-gel and co-precipitation methods, further corroborating our findings.

In terms of bioprocess optimisation, the volume of the harvesting medium significantly affected L-dopa production. A reduction in yield was observed beyond 75 mL, likely due to decreased oxygen availability and agitation efficiency. These results are consistent with Mariam et al. (2010), who reported maximum production at 50 mL of culture medium. Conversely, Haq et al. (2002) found the highest L-dopa yield at 25 mL, highlighting the strain-specific nature of fermentation efficiency.

The effect of pH on L-dopa production showed optimal results at pH 6.5, likely due to enhanced metabolic and enzymatic activity. This aligns with Ali and Haq (2010), but contrasts with Surwase et al. (2011), who reported maximum L-dopa production at pH 8 using *Brevundimonas* sp. The difference could be attributed to microbial variation. Agarwal et al. (2016) also reported optimal L-dopa synthesis at pH 5.5–6.0, while Olsen (1981) observed peak yields at even lower pH (4.0), further indicating that optimal pH is species-dependent.

The incubation time also had a marked effect on L-dopa synthesis. Maximum production was recorded at 48 h, beyond which a decline occurred, possibly due to nutrient depletion or microbial overgrowth. These findings are supported by Raju et al. (1993), who also identified 48 h as the optimal incubation period. However, Surwase et al. (2011) found optimal L-dopa yield at 18 h, while Haneda et al. (1973) reported it at 72 h, again reflecting the influence of microbial strain and cultivation parameters.

Kinetic parameters indicated higher V_max_ and K_m_ values of immobilized tyrosine hydroxylase with K_cat_ value of 1.40 × 10^18^ s^− 1^. The kinetic parameters K_m_ and V_max_ for immobilized tyrosine hydroxylase (TH) vary depending on the immobilization method and the specific conditions of the experiment. Kinetic parameters for TH from *Bacillus subtilis* are not exactly reported. Tyrosine hydroxylase (from rat), for example, exhibited a K_m_ of 108 microM and a V_max_ of 6.93 mumol/min/mg of catalytic subunit (Roskoski and Ritchie 1991). K_cat_ value of 1.48 × 10^18^ per second for immobilized TH indicates that each enzyme molecule can convert approximately 1.48 × 10^18^ substrate molecules into product molecules every second when the enzyme is saturated with substrate. This high value signifies an extremely efficient enzyme, capable of catalyzing a large number of reactions very rapidly. Significant reusability of immobilized with 71.88% retention in activity after five cycles is in accordance to Khan et al. (2025).

## Conclusion

The present study investigated the production of tyrosine hydroxylase (TH) from *Bacillus subtilis* SDSC-Env-i6 and its surface conjugation with aluminium oxide (Al₂O₃) nanocrystals. The results indicate a reduction in aromatic L-amino acid decarboxylase (AAA decarboxylase) activity by approximately 4.4-fold and 3.6-fold in the presence of benserazide and carbidopa A, respectively. Optimal enzyme stability was observed at 30 °C with carbidopa and 35 °C with benserazide. The highest tyrosinase activity was achieved using 15 ppm CaCl₂ (6.3 U/mL) and CuCl₂ (5.5 U/mL). Notably, enzyme conjugation with Al₂O₃ nanoparticles resulted in up to a five-fold increase in activity compared to the free enzyme, with peak activity (15.3 ± 0.05 U/mL) recorded under optimised experimental conditions. SEM analysis confirmed the formation of irregular spherical Al₂O₃ nanoparticles ranging from 124 to 130 nm in size. These findings are truly supported by significant kinetic parameters and enzyme turn over values. While these findings suggest potential for industrial application, particularly in the context of L-dopa production, the results should be interpreted with caution. Future research should aim to validate L-DOPA production using the conjugated enzyme under application-relevant conditions and assess long-term stability, biocompatibility, economic feasibility and enhanced reusability at scale.

## Data Availability

Data are available on request.
